# Compound tool construction by New Caledonian crows

**DOI:** 10.1038/s41598-018-33458-z

**Published:** 2018-10-24

**Authors:** A. M. P. von Bayern, S. Danel, A. M. I. Auersperg, B. Mioduszewska, A. Kacelnik

**Affiliations:** 1 Department of Zoology, University of Oxford, OX1 3PS Oxford, UK; 2 Department II of Biology, Ludwig-Maximilians-Universität München, 82152 Planegg-Martinsried, Germany; 3 Max-Planck-Institute for Ornithology, 82319 Seewiesen, Germany; 4 Laboratory for the Study of Cognitive Mechanisms, University of Lyon, Bron Rhône-Alpes, 69500 France; 5 University of Veterinary Medicine Vienna, 1210 Wien, Austria; 6 Messerli Research Institute, Medical University of Vienna, 1010 Wien, Austria; 7 Messerli Research Institute, University of Vienna, 1090 Wien, Austria

## Abstract

The construction of novel compound tools through assemblage of otherwise non-functional elements involves anticipation of the affordances of the tools to be built. Except for few observations in captive great apes, compound tool construction is unknown outside humans, and tool innovation appears late in human ontogeny. We report that habitually tool-using New Caledonian crows (*Corvus moneduloides*) can combine objects to construct novel compound tools. We presented 8 naïve crows with combinable elements too short to retrieve food targets. Four crows spontaneously combined elements to make functional tools, and did so conditionally on the position of food. One of them made 3- and 4-piece tools when required. In humans, individual innovation in compound tool construction is often claimed to be evolutionarily and mechanistically related to planning, complex task coordination, executive control, and even language. Our results are not accountable by direct reinforcement learning but corroborate that these crows possess highly flexible abilities that allow them to solve novel problems rapidly. The underlying cognitive processes however remain opaque for now. They probably include the species’ typical propensity to use tools, their ability to judge affordances that make some objects usable as tools, and an ability to innovate perhaps through virtual, cognitive simulations.

## Introduction

Tool-related behavior, especially innovative tool manufacture, is intimately associated with human evolution, and may have co-evolved with specific neurological capacities, particularly planning and complex task coordination^1^. Innovative tool manufacture emerges only between 5–9 years of age in human ontogeny^2–4^, probably because inventing new tools requires cognitive operations that include executive functions^5^ that develop only late^6^ and after extensive individual and social learning. Outside humans, innovative tool manufacture is only known in a small set of taxa, notably in other primates, corvids and parrots (e.g.^7–13^).

Here we focus on a particularly rare form of tool innovation, a type of tool manufacture that anthropologists and primatologists consider profoundly significant for understanding human evolution^1^. Creating maneuverable tools by combining complementary different parts into firmly connected units referred to as compound tools hereafter (also labeled composite tool manufacture^1^ or additive tool making^14^), is a particularly rare form of tool manufacture, hitherto unproven outside the hominid lineage. Even among hominids, innovative compound tool construction has been documented only through a few reports in captive great apes^7,8,10,13,14^ and some authors consider it to be absent in wild chimpanzees (*Pan troglodytes*)^15,16^. The latter do, however, display behavior that involves combining different elements into functional assemblages. At Bossou, Guinea, for example, some individuals combine 3 or more elements: they stabilize a relatively large planar rock using a second stone as a wedge, and then use a third stone as a hammer to pound nuts^17^. The components have specific roles (anvil, wedge, hammer) and are chosen and placed appropriately to make the assemblage functional, but this assemblage forms a tool and substrate composite rather than a compound tool, which is an integrated mobile object^14^. In another example, at the Sonso community in Budongo Forest, Uganda, wild chimpanzees hold together multiple leaves and use them as sponges to extract water or honey from cavities^15^. The bundles of leaves are jointly mobile, but they form loose, amorphous aggregates of equivalent parts, and it is debatable whether, for the purpose of understanding tool-related cognition, it is useful to integrate them in the category of compound tools. How such tool technologies are discovered and adopted by individual members of different natural populations is an important but as yet largely unsolved problem. In particular, the relative roles of genetic predispositions, individual innovation and social transmission are not easy to establish, because in wild populations all those factors contribute and are confounded from the observer’s perspective. In captivity in contrast, the acquisition process of complex novel behaviors such as compound tools can be studied and manipulated under controlled conditions.

The best-known case of the invention of a compound tool outside humans was reported in captivity nearly a century ago by Koehler^7^. He reported that a captive chimpanzee (Sultan) discovered how to combine 2 pieces of bamboo so as to make a longer compound pole with which he could reach bananas placed outside his cage^7^. Koehler’s observation is of major significance for comparative cognition, because it seems reasonable to hypothesize that for an individual to invent an instrument by joining different objects into a novel structure, anticipating its emergent properties (affordances) the individual must be capable of some form of cognitive modeling. Koehler, and many of his followers, have used the term insight in an explanatory role, but, in the absence of precise controlled experimentation and working definitions, such as used in human psychology (e.g.^18,19^), this does little more than labelling the observed sudden behavioural transition. His seminal observation, while frequently cited, has remained underexplored in later comparative cognitive research (See^10^ and^13^ for exceptions), and has not yet been extended to taxa beyond primates.

We were inspired by Koehler’s study to investigate compound tool inventions, but in the absence of social inputs, previous experience with similar tasks, or reinforcing feedback, in New Caledonian crows, a species notable for exhibiting multiple flexible and species-specific tool-related abilities^20–24^. Laboratory experiments have shown that these crows possess a capacity to innovate^25–27^ and to solve novel physical problems with sensitivity to at least some causal physical interactions between objects^28,29^. Given what is known about this species, it seems a strong candidate to explore its competence for compound tool making. Should this be successful, it would provide a novel and independently evolved biological model^30^ to help understanding the associated cognition.

**Figure 1:**
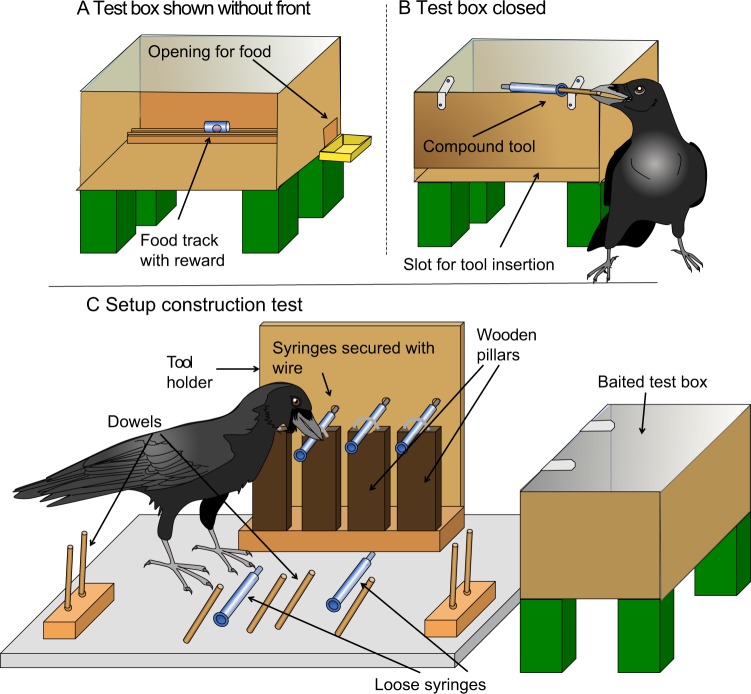
Experimental setup in construction test 1. Upper panels: test box without (**A**) and with (**B**) front cover. Notice the food track and side opening in A, and the narrow slot for tool insertion in the front in B. (**C**) Presentation of tool components. Some details of scale modified for presentation (see SI for details).

The present study presented 8 wild-caught New Caledonian crows (NCC hereafter), with a problem solving task they had never encountered before. The study includes 5 phases: (i) set-up familiarization, (ii) first construction test, (iii) transfer tests (involving 2 modifications of the task), (iv) need discrimination test and (v) second construction test. In the set-up familiarization phase the subjects were presented with a novel setup, i.e. food placed in a track inside a transparent box, and wooden doweling pieces of sufficient length for them to push the reward along this track and out of the box (Figs 1 and S1). After the crows had successfully extracted the food, the first construction test followed, where the long dowels were replaced by 2 novel kinds of cylindrical elements, all too short to reach the food. These elements were potentially combinable, one kind being hollow (1 ml syringe barrels) and the other solid and thinner (syringe plungers or short wooden dowels; see methods and Supplementary Information, SI hereafter, for further details). They were presented both on holders and scattered on the ground (Figs 1 and S2). Subjects were allowed up to 6 attempts (of max. 12 min each) to retrieve the food. Successful birds entered into the third phase, that tested whether the production of compound tools depended on a particular shape and configuration of the combinable elements. This phase involved 2 transfer tests. In the first, subjects were exposed to novel materials; pipe cleaners were provided in addition to wooden dowels and the syringe barrels were replaced by drinking straws presented in a novel position, as well as loosely on the ground (Fig. S3). In the second, the ergonomic difficulty was increased by presenting all the straws and dowels solely on the floor (Fig. S4).

The fourth phase was designed to test whether the birds constructed tools in a goal-directed manner, driven by the need for compound tools, or simply because the action of combining was rewarding by itself. To this effect a *‘close track’* was added, from which a food target could be retrieved with uncombined elements (Fig. S5), and alternated between this situation and the original track, now called ‘*distant’*. After familiarization with both short and long tools (uncombinable dowels) simultaneously available in the presence of either of the tracks (see SI for details), we provided only short, combinable, tool elements, and positioned the food half of the trials in the distant and half in the close track, in pseudo-random order. Finally, in the fifth phase, the flexibility and goal-directedness of the behavior was examined by further challenging the birds that had succeeded in making stable 2-elements compound tools and extracting food with them in all previous phases. Now they were supplied with even shorter tool elements, so that 2-component tools were insufficient to extract the food (SI; Fig. S6) making an additional recursive combination necessary.

## Results

### Set-up familiarization

All 8 birds readily used the provided long dowels to extract the food within their first trial.

### Construction test 1

Four of the 8 crows succeeded in extracting the reward by joining 2 elements into a single, longer mobile pole. They did so within a very short time (less than 4–6 minutes of interacting with the parts; see Tables 1 and S1 for details) and with no apparent trial and error learning, i.e. choosing suitable elements and taking no or few unrewarded attempts (Tumulte: 0, Tabou: 3, Mango: 7 and Jungle: 10; see Table S2) before successfully combining them. The successful birds acted in a seemingly purposive manner, using the compound tools to aim at the food immediately after creating them (Movie S1,2). This typically required transporting the compound to the box for use. Except for one individual, Mango, a bird with apparently fluctuating motivation, the successful subjects also solved the task in the 3 opportunities that followed their first success (see SI). Mango refused to participate in 2 follow-up trials but succeeded continuously afterwards.

**Table 1:** Individual path to successful use of compound tools.

Successful subjects	Construct. test (syringe)			Transfer test 1 (straw)			Transfer test 2 (ground)		
	interaction time (min:sec)	1^st^ comb. attempt	1^st^ succ. (trial nb.)	interaction time (min:sec)	1^st^ comb. attempt	1^st^ succ. (trial nb.)	interaction time (min:sec)	1^st^ comb. attempt	1^st^ succ. (trial nb.)
***Jungle***	5:33	1	**3**	8:22	1	**6**	0:32	/	**1**
***Tumulte***	4:42	/	**2**	0:14	/	**1**	2:48	3	3*
***Tabou***	5:08	3	**6**	8:26	3	**4**	4:14	1	**3**
***Mango***	3:55	1	**5**	0:39	/	**2**	4:32	/	**1**

Summary of performance in construction test 1 (‘syringe’) and the 2 transfer tests (‘straw’ and ‘ground’) for the 4 subjects that made and used compound tools. The table shows total interaction time with the tool elements before success (interaction time); trial number of the first combinatory attempt (1^st^ comb. attempt); and trial of first successful construction and use of a compound tool (1^st^ success). Diagonal slashes denote success at first attempt (without prior failures). The interaction time until first success were re-scored by a second rater to check inter-observer reliability. The Pearson’s correlation coefficient was more than 99% (Pearson’s correlation coefficient: *r* = 0.993, *p* < 0.001). *Identifies a trial in which a subject constructed an unstable compound tool.

Individuals differed in their path to success (see Tables 1 and S1 for details), possibly due to differences in motor ability and/or motivation, as well as in cognition, but none of the individual trajectories point to the behavior emerging through gradual shaping by reinforcement of random actions. The 4 unsuccessful birds continued to aim at the food with the too-short elements supplied, hardly ever making any attempt to combine them.

### Transfer tests 1 and 2

In Transfer test 1, all 4 birds made and used compounds with no more than 2 combinatory attempts prior to success (see SI, Tables 1, S1 and S2 and Movie S3). In Transfer test 2, all 4 birds made compound tools in less than 5 minutes of interaction, (see SI, Tables 1, S1 and S2 and Movie S4). Two crows made the compound and extracted the food in their very first trial, and the remaining 2 made their first compounds in their third trial, but one of them (Tumulte) failed to retrieve the target because the compound it built disintegrated during the attempt.

### Need discrimination test

**Figure 2:**
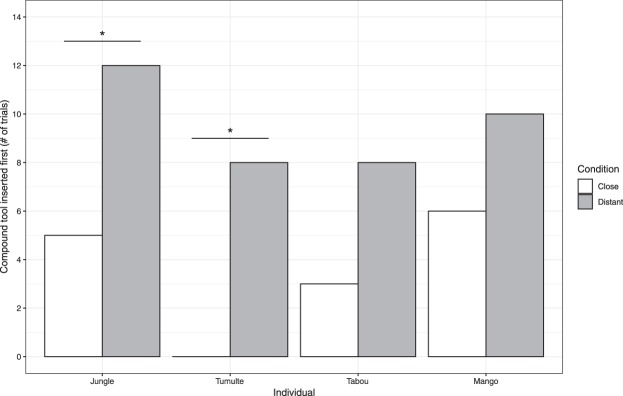
Performance in the need discrimination test. The y-axis shows the number of trials (out of 12), in which compound tools were the first to be inserted in the close (blue bars) and distant (red bars) track conditions. Asterisks indicate p < 0.05 in Fisher’s exact test (Fisher’s p = 0.037 for Jungle, 0.001 for Tumulte, 0.100 for Tabou and 0.193 for Mango).

All 4 birds tried with an uncombined tool first more often when food was in the close than in the distant track (means 70.8% and 20,8% respectively (Fig. 2). Put another way, they made and used compounds without trying at all with uncombined elements first on 79.2% of cases when it was necessary (i.e. food in the distant track), and only 29.2% of cases when it was not (i.e. food in the close track). For 2 birds the results were within conventional significance (see Fig. 2). Even though all birds showed rather strong effects, the fact that only 4 birds reached this stage precludes a statistical evaluation at group level.

### Construction test 2

In this final phase a 2-piece tool was not long enough to reach the food, so multiple pieces had to be combined to succeed. One individual (Mango) repeatedly created and used tools made of 3 and 4 elements (movies S5 and S6). The other 2 crows readily created 2-element tools and tried to use them, but failed in the additional recursive step that was now required. They did not make any combinatory attempts beyond non-functional 2-element compound tools. Mango in contrast made the first combinatory action between a 2-component tool and a third element from trial 1, but had difficulties in adding on the third element with enough force to achieve a tool of sufficient stability. From trial 4 onwards and after a total of 7 combinatory actions, he produced stable and usable 3-component tools. Specifically, this subject built 3-compound tools with which he successfully extracted the food in trials 4, 5, 6, 7 and 10 (also employing a 3-component tool in trial 9, but failing to extract the food) and created a 4-compound tool in trial 6 and 9, which he employed in the box moving the food. He also built several 3-component tools that had the appropriate length but fell apart during insertion in trials 4 and 6 and additionally a 4-compound tool, which he did not use in trial 11 (see SI for additional details).

## Discussion

The finding that a bird species has the capability to rapidly discover how to construct novel, functional tools through assemblage of different, otherwise non-functional, parts, matches and exceeds present evidence for this ability in non-human primates^7,8,10,13,14^.

In Koehler’s emblematic study^7^, the male chimpanzee, Sultan, made a useful compound pole, but only after being coached by a human demonstrator who poked his finger into the hollow bamboo element. According to Koehler, Sultan manipulated the tool elements for over an hour and then, after a short break (not long after the aforementioned demonstration), suddenly discovered the solution, as if overcome by an acute insight. In contrast, half of our 8 crows succeeded, similarly abruptly, but within only 4-6 min of engaging with the tool elements, and without any cueing by the experimenter. Also, Sultan did not immediately reproduce the constructive behavior the following day, while 3 of our 4 successful crows readily continued to produce compound tools in the trials that followed their first occasion. They also transferred to modified situations rapidly and demonstrated sensitivity to the need for tool construction.

Although, operationally defined, the crows achieved a “tool innovation”^3,4^, because they succeeded in creating a novel compound tool (i.e. a type of tool not found in wild populations and one the subjects had never encountered before) as response to a specific novel problem, the cognitive processes involved remain opaque for the moment, and there is no reason to assume that the cognitive operations leading to compound tool constructions are identical in apes and birds. Labeling the sudden transition to constructing tools as an “insight”, as Koehler and many followers did, does not further elucidate those processes. The question to address is whether the crows invented the compound tool, i.e. whether they came up with the solution as the result of a creative process that potentially involved some form of virtual mental modeling (i.e. simulating operations and motor actions with neural representations of the real objects), or whether they constructed their first compound tools accidentally, and then adopted it through reinforcement learning. Both paths to acquisition would be interesting, and the latter would in principle be a preferable explanation because, prima facie, it relies on simpler and better-known processes. However, while we do not have any algorithmic proposal for how cognitive operations based on representations of the participant objects can be accessed, the reinforcement learning route seems to fall short of being able to reproduce the birds’ innovative behaviour.

Let us consider in detail the possibility that the successful birds produced their first compound accidentally, i.e. searching for food inside the hollow short element using the solid stick-like elements as poking tools, rather than in an attempt to combine them in order to use the resulting compound tool in the target box. Such probing could indeed occur, since New Caledonian crows do have heritable predispositions to use stick-like tools for extractive foraging and exploration^22,31,32^. Their natural foraging behavior includes inserting sticks into natural orifices in substrates, aiming tool tips precisely at targets in those holes^33,34^. If the short pieces accidentally stuck together during food probing, the subject might have subsequently recognized them as a sufficiently long tool to reach for the food target in the box, and reinforcement could lead to an increase in the probability of combining from then on. Judging distances and required lengths of tools, as well as choosing, making and modifying tools accordingly, are all capabilities already known in this species^35,36^. One thing that speaks against this possibility, however, is that the crows’ food searching was very obviously focused on the box with the visible bait rather than the hollow elements. The 4 successful birds inspected the box persistently and ostensibly from various sides, repeatedly trying to reach for the reward with short single elements. Only in brief interruptions of such food reaching bouts, did they attend to the hollow elements and when they succeeded in making a compound they invariably took it to the baited box and used it to reach the food. This persistence in attempts to extract a detected but unreachable food item is a typical behaviour in the species (e.g. *36)*. Further, the topography of their behavior when aiming short solid elements into hollow ones did not resemble what they do when searching for food: rather than producing rapid up and down probing movements^20^, the crows made a single or very few controlled insertions, steadily pushing the 2 elements into each other with substantial force and, once the elements stuck together, carefully pulling the compound out (Movies S1–4) and transporting it to the food box.

In trials that followed a first construction the birds constructed compounds immediately, and in the two transfer tests in which the available short elements were different, they rapidly used the novel elements to build compounds. In the need discrimination test in which tool construction was only required in half of the trials. All four successful birds built compounds without previously poking with uncombined elements more often when construction was necessary than when it was not, even if construction had by that stage been reinforced repeatedly (Fig. 2). This difference however was significant for only 2 of them.

Finally, one of the 3 tested individuals succeeded to manufacture tools out of 3 and even 4 elements. This multi-compound tool construction required dexterity and perseverance. It involved both combining hollow elements with sticks and the other way around, as well as turning the tool to insert the solid end in another hollow element. Accidental discovery of this recursive process (treating a 2-element compound as a potential part for further combination and construction of a 3-element one, and so forth) seems implausible. To our knowledge, this is the first evidence of compound-tool construction with more than 2 elements in any non-human animal. It would appear that at least for one of the birds (Mango), the challenge of making multiple (>2) component tools was ergonomic rather than cognitive: he made his first combinatory attempts with 3 elements from the first trial onwards, thus demonstrating sensitivity to the need for the next recursive step, but took a few trials to make them sufficiently stable to be operable. This was not the case for the other 2 subjects tested in this task. While readily producing 2-compound tools, they did not make any combinatory attempts with a third element.

Thus, in summary, for a variety of reasons the behaviour of the successful subjects argues against accidental discovery, or gradual trial and error learning through reinforcement. The alternative is less parsimonious as it appeals to ‘higher’ cognitive processes that have not yet been modeled explicitly in either natural or artificial systems. Developing such explicit models is highly timely, as recent comparative cognition research is revealing that different animal species exhibit abilities to solve novel problems beyond the scope of reinforcement learning. Indeed, our own experimental species, the NCC, has been shown to possess other remarkable innovative tool-related problem-solving abilities in the laboratory, such as spontaneous sculpturing of novel materials into functional tools^9,37^, using novel types of tools^25,27^ and causal information to solve problems^28,29^ and employing several tools in a sequence to reach a goal^24,36^ - all in the absence of reinforcement or cueing. Their compound tool construction constitutes a new example of their ability to generate solutions to novel challenges, and corroborates that their abilities are highly flexible and subject to individual variation. The hard challenge ahead is the development and testing of algorithmic models capable of similar performance, whether in silico or in artificial physical systems.

## Methods

### Subjects and housing

Subjects were 8 wild-caught, captive, adult New Caledonian crows (*Corvus moneduloides*), 4 females (Liane, Tortue, Tumulte, Tabou) and 4 males (Jungle, Mango, Aigaios and Papaye) which had a minimum age of 3 years. They had been wild-caught in 2010 and, since then, were kept at the Avian Cognition Research Station of the University of Oxford, UK, hosted by and associated to the Max-Planck-Institute for Ornithology, Germany. The crows were housed in pairs in spacious outdoor aviaries with adjacent heated indoor areas, kept at a 12:12 h dark-light regimen (for more details see SI) and had *ad libitum* access to food and water. The subjects were naïve in respect to the problems presented here, but familiar with the use of simple tools for extractive purposes, which forms part of their natural behavioral repertoire^20,22,31^.

### Basic Apparatus

Testing took place in the indoor enclosures in visual isolation from their mates. The basic apparatus was set up on the ground, and consisted of a partly transparent test box that contained a food reward (Figs 1 and S1) placed in a track that run parallel to the box’ front at 15 cm distance. At one end of the track there was a hole through which the bait could be extracted. Different tool elements were provided on different holders and loosely, scattered on the ground, depending on the condition (see Figs 1, S2, S3 and S4). They consisted of syringe barrels or drinking straws, which were ca. 8 cm or 5 cm long and could be combined with ca. 8 cm or 5 cm long solid elements of smaller diameter (wooden dowels, syringe plungers, pipe cleaners) into compound tools. The hollow elements were fitted with a clot of children’s modeling clay at ca. 1 cm from the opening for the syringes and from both openings for the straws so that they could consolidate with the counterparts when firmly pushed into each other (for more details see SI).

### Setup and Procedures

#### Setup familiarization

The experimental series started with a 3-hour period of familiarization, during which the subjects were exposed to the test box and both tool holders (see Figs S1, S2 and S3) in the indoor enclosures without any tools, tool elements or rewards present, to overcome any initial neophobia. The next day the actual setup familiarization phase followed, during which subjects could experience the functionality of the box. Here, again the test box was present with both empty tool holders and without any tool elements, but now a reward was placed in the food track in the box. and wooden dowels (ca. 15 cm) sufficiently long to reach it were provided. The birds could insert the dowel into the box’s front slot and use it to slide the food along the track, until it fell out of the box through a side opening. Each bird passed to the next stage after it had succeeded at least 6 consecutive times.

#### Construction test 1

In this phase 2 types of potentially combinable 8 cm long tool elements were provided instead of the 15 cm long dowels, thus the food was now beyond the reach of single elements, which the birds had not experienced before. The setup consisted of the box and the syringe tool holder (Fig. S2) equipped with 6 hollow elements (1 ml syringe barrels; syringes thereafter) and 8 solid, thinner elements (syringe plungers, soon substituted by wooden dowels for practical reasons; see SI for further detail) presented on holders and laid out on the board (see Fig. 1 for a graphic representation). Subjects received up to 6 trials that lasted up to 12 min each. Once a subject succeeded in making a compound and extracting the food it participated in “replication trials” to test whether it would readily reproduce its success, until it had either succeeded in 3 or failed in 6 consecutive such trials.

#### Transfer tests 1 and 2

The 4 successful birds were presented with 2 new scenarios, where different, but still combinable 8 cm elements, were dispensed in novel positions and/or loosely on the ground. In transfer test 1 the setup included a straw holder with 6 drinking straws, 8 short dowels and 8 short pipe-cleaners, presented on holders at a 45° angle and laid out on the board, as shown in Fig. S3. Subjects received at least 5 trials that lasted up to 12 min each. In Transfer test 2 all potential tool elements (6 straws, 6 syringes and 8 dowels) were placed on the ground (Fig. S4). To avoid introducing unnecessary changes, the (empty) tool holders were left in place. The trials lasted max. 12 min and each bird received at least 8 trials. As in construction tests, subjects that successfully created compound tools received replication trials.

#### Need discrimination test

In this test, trials differed in whether a compound was necessary or not. The position of the food now varied pseudo-randomly between a new “close track” positioned 6 cm from the box’s front and the original, *distant* track at 15 cm distance from the front. Targets could be retrieved from the close track with uncombined elements, i.e. without any constructive action (Fig. S5). We first familiarized the birds with the food being either in the *close* or in the original *distant* track, with both short and long tools (uncombinable dowels; 8 cm and 13 cm respectively) simultaneously available (for details see SI materials and methods section). After familiarization, we provided only short (8 cm), combinable tool elements (6 straws, 6 syringes and 8 dowels), and positioned the food either in the close or in the distant track, in pseudorandom order until completing 12 trials at each position (8 sessions of 3 trials). The setup was the same as in transfer test 2, i.e. the tool elements were presented loosely on the boards, leaving the 2 holders (see Fig. S4) themselves empty. For the subject that had failed in transfer test 2, tool elements were additionally provided on the 2 holders, as in the construction test 1 and transfer test 1 respectively. We focused our analysis on the first attempt to retrieve food by inserting a tool in the box, to exclude cases in which a bird tried a short element even though a compound was needed and only after failing opted for construction.

#### Construction test 2

The 3 birds that succeeded in making stable tools and extracting food in transfer test 2 (i.e. excluding Tumulte, that had made a compound but failed to extract food) were tested with even shorter tool elements (ca.4,5–5 cm) so that a 2-component tool was insufficient to extract the food in the track at 12 cm distance from the box’s front (Fig. S6). A new syringe tool holder consisting of a board with 4 pillars that loosely held short (ca. 4,5–5 cm) ‘syringe tubes’ (each made out of 2 syringe ends glued together) was set up next to or perpendicular to the original test box. Two additional short syringe tubes and 10 short (ca. 5 cm) dowels were also presented as shown in Fig. S6. Functional tools could be obtained by combining 3 tool elements, either using a syringe tube with a dowel at either end, or a dowel with a syringe tube at either end. Twelve trials of max.12 min each were conducted.

### Analysis

Video analyses were carried out to score individual performance, frequency of combinatory attempts (trying to insert a tool element into the opening of another, or inserting a tool element into another, but without succeeding to create a combined tool sufficiently stable to be lifted) and interaction time with tool elements (time in sec an individual spent manipulating/touching tool elements until first success). A trial was scored as successful if a subject created a sufficiently stable combined tool, inserted it in the test box, and retrieved the food. The data were scored by two raters independently who had absolutely no discrepancies in scoring instances as successful compound tool construction or not. Additionally, inter-observer reliability was assessed for the interaction time until first success and the Pearson’s correlation coefficient was more than 99% (Pearson’s correlation coefficient: *r* = 0.993, *p* < 0.001).

For the need discrimination test, where food was located either within or beyond the reach of uncombined single tool elements, we examined whether the birds first inserted a combined or uncombined tool into the test box in each of the 12 trials of both conditions (*close* and *distant* track), and also looked at whether individuals successfully retrieved the food with a combined or uncombined tool. Because we had only 4 subjects for this test, we evaluated the statistical reliability of the results with Fisher Exact tests at individual level.

### Ethical approval

All applicable EU, national, and/or institutional guidelines for the care and use of animals were followed. No specific permissions were required under German law (§7 Bundestierschutzgesetz) for this non-invasive study. The methods were approved by the ethics committee for research not involving invasive procedures of the Zoology Department, University of Oxford.

## Electronic supplementary material

Supplementary Information

Movie S1

Movie S2

Movie S3

Movie S4

Movie S5

Movie S6

## Data Availability

Exemplary videos can be accessed in the SI. The datasets analysed during the current study are available from the corresponding author on reasonable request.
