# Supplementary material for: Compound tool construction by New Caledonian crows
Source: Sci Rep. 2018 Oct 24;8:15676. doi: 10.1038/s41598-018-33458-z (PMC6200727; doi:10.1038/s41598-018-33458-z)
Supplement: Supplementary file 1 — Supplementary Information [file 41598_2018_33458_MOESM1_ESM.pdf]

Supplementary Information:

Compound tool construction by New Caledonian crows

A.M.P. von Bayern[1,2,3,]*, S. Danel[3,4], A.M.I. Auersperg[5], B. Mioduszewska[2,3], A. Kacelnik[1,]*

Author affiliations:

[1]Department of Zoology, University of Oxford, OX1 3PS Oxford, UK.

[2]Department II of Biology, Ludwig-Maximilians-Universität München, 82152 Planegg-Martinsried, Germany

[3]Max-Planck-Institute for Ornithology, 82319 Seewiesen, Germany.

[4]Laboratory for the Study of Cognitive Mechanisms, University of Lyon, Bron Rhône-Alpes 69500, France.

[5]Messerli Research Institute, University of Veterinary Medicine Vienna, Medical University of Vienna, University of Vienna, 1210 Wien, Austria.

*Correspondence to: avbayern@orn.mpg.de and alex.kacelnik@zoo.ox.ac.uk.

This PDF file includes

A. Extended Materials and Methods

B. Extended Results

C. Figures S1-S6

D. Tables S1-S3

E. Movies S1-S6

A. MATERIALS AND METHODS.

1. Subjects and Housing.

Subjects were eight wild-caught captive New Caledonian crows (*Corvus moneludoides*), four females (Liane, Tortue, Tumulte, Tabou) and four males (Jungle, Mango, Aigaios and Papaye) of a minimum age of 3-6 years (based on gape coloration estimates at the time of capture). The birds were wild-caught and kept at the Avian Cognition Research Station of the University of Oxford, U.K., hosted by and associated to the Max Planck Institute for Ornithology, Germany. They were housed in large and enriched outdoor aviaries (ca. 25m^2 and 2,5m height) with adjacent heated indoor rooms and experimental compartments (ca. 5m^2 and 2m height) lit with flicker-free Arcadia® bird lamps with a sunlight UV spectrum, and a 12:12h dark-light cycle. Six of the crows were kept as pairs, while one female (Tortue) was housed alone for future pairing and one male (Aigaios) was group-housed with its parents. Fresh water and a diet consisting of a varying meat mixture (minced meat, minerals and vitamins, curd, egg, dried

insects, boiled rice, and vegetal oils), cereals, fruit, soaked cat biscuits, and Versele Laga® Beo pearls were available *ad libitum*.

2. Experimental Apparatus and Setup.

Testing took place in the indoor enclosures of the home cages, which were previously cleared of all potential tools. Birds were visually separated from their mates for the duration of each trial. The test box (figure S1) and a flat board with the tool holder(s) were set up on the ground, before the subject entered the indoor test aviary. Tool holders for the first construction test and the first transfer test are shown in figures S2 and S3.

At the start of each trial the food container was placed in the food track inside the test box, at least 15cm from the side opening (figure S1) so that it could not be reached through the side. The test box was placed with its back near a wall of the indoor testing compartment, while the board with the tool holder(s) was placed to its left, at a 90° angle at ca.7cm distance, or - depending on shape of the aviary - aligned to the front of the test box, i.e. without the perpendicular angle to it. If 2 tool holders were present (e.g. during the setup familiarization phase and in transfer test 2), they were placed as shown in figure S4).

Different tool elements were provided on the tool holders and/or on the boards, depending on the condition. They consisted of syringe barrels (syringes thereafter) or drinking straws (straws thereafter), which were ca. 8cm long and could be combined with ca. 8cm long solid elements of smaller diameter (wooden dowels or syringe plungers* and pipe cleaners) into compound tools of ca. 14cm length. This length sufficed to reach the reward 12cm away from the front of the box (figure S1). The hollow elements were fitted with a clot of fresh, soft children's modeling clay at ca. 1-1,5cm from the opening for the syringes and from both openings for the straws, so that they could fuse with the solid counterpart when firmly pushed into each other.

During the set up familiarization phase, both tool holders (figures S2 and S3) were present, together with the test box but without any tool elements. Instead long sticks were provided, with which the food could be reached.

During later testing, the test box was always present but the remaining setup varied depending on the condition:

1. *Setup in the construction test 1:*

The setup consisted of the syringe tool holder equipped with 6 1ml syringe barrels (syringes thereafter) and 8 dowels (/syringe plungers) presented on holders and laid out on the board as shown in figure S2.

2. *Setup in the transfer test 1 (straw):*

The setup included a straw holder with 6 drinking straws, 8 short dowels and 8 short pipe-cleaners, presented on holders and laid out on the board, as shown in figure S3.

---

* Plungers were exchanged for wooden dowels (and a clot of modeling clay was added to the syringes so that they could be joined with wooden dowel counterparts) after the first 1-3 trials of the 1st construction test, because one of the crows removed the rubber plunger tip that was essential for the plunger to stay stably joined to the syringe if inserted partially to produce a long tool. This particular trial was disregarded. One bird, Papaye, only reached training criterion after this happened and was tested with dowels from the beginning.

3. Setup in the transfer test 2 (ground):

In this case all potential tool elements (6 straws, 6 syringes and 8 dowels) were placed on the ground as shown in figure S4. To avoid introducing unnecessary changes, the (empty) tool holders were left in place.

4. Setup in the need discrimination test:

The syringe and straw tool holders and the test box were set up as in transfer test 2. However, the test box now had an additional food track and side opening, both closer to the tool slot, at ca. 6cm distance from the box's front ('close' track; see figure S5). For the 3 individuals that had succeeded in transfer test 2 (i.e. the ground condition) tool elements (6 straws, 6 syringes and 8 dowels) were presented as in that test, i.e. loosely on the boards, leaving the holders themselves empty. For the subject that had failed in transfer test 2, tool elements were additionally provided on the 2 holders, as in the construction test 1 and transfer test 1 respectively.

5. Setup in the construction test 2 (three-component tool):

A new syringe tool holder (figure S6) consisting of a board with 4 pillars that loosely held short (ca. 4,5cm) 'syringe tubes' (each made out of 2 syringe ends glued together) was set up next to or perpendicular to the original test box. Two additional short syringe tubes and 10 short (ca. 5cm) dowels were also presented as shown in figure S4. The syringe tubes could serve as connections between 2 dowels, thanks to their two wide openings facing outwards. Alternatively, one dowel could connect 2 syringe tubes.

3. Experimental Procedures.

1. General and Functional Setup Familiarization:

The experimental series started with a period of general familiarization, during which the subjects were exposed to the test box and tool holders (figures S1 and S2) without tools or reward for 3 hours, to overcome any initial neophobia. The next day the functional setup familiarization phase followed, during which subjects were allowed to experience the functionality of the test box. Here we provided long dowels (ca. 15cm) with which the birds could reach the reward on the food track. All subjects quickly discovered how to retrieve the bait by inserting the dowel in the front slot and sliding the reward along the food track towards the opening at its end on the left or right side, until the food dropped onto the food plate. Each bird passed to the next stage after it had succeeded in retrieving the bait at least 6 times in a row.

2. *Construction test 1 and transfer test 1 (straw):*

In these 2 phases the birds faced the baited box as before, but now without tools of sufficient length to reach the target. Potential tool elements were offered as shown in figures S2 and S3. Sufficiently long tools could be made by combining a dowel/plunger with a syringe (construction test 1) or a dowel/pipe cleaner with a straw (transfer test 1). All the birds were given at least 6 construction trials with syringes (figure S2) and 5 transfer test 1 trials with straws (figure S3), so that each bird had at least 11 tests of 12 min duration, unless it retrieved the food before the 12 minutes had lapsed, in which case the trial was stopped. The action was monitored by surveillance cameras (and recorded on camcorders). Subjects that succeeded in the construction test faced replication trials (see below) before continuing.

Procedures applying to the construction test(s) and the transfer tests:

Subjects participated in 2-4 trials per day, spaced apart as much as logistically possible, so that intervals of 2-8hrs lay between them. There were 2 exceptions: for 2 subjects (Mango and Jungle) more trials (5 and 6 respectively) were conducted during one testing day during the 2nd transfer test (ground) due to time constraints, so that the inter-trial-intervals on that day were only a minimum of 1,5hrs long. We dismissed trials when the bird did not touch or approach to closer than 20cm of the test box, or when it accidentally found in the aviary an unplanned (natural) tool of sufficient length to reach the food. Although the experimental room was cleared of potential tools prior to each test, on a few times tools were overlooked because the birds had well-hidden tool caches.

Replication trials: In order to see whether the crows could readily reproduce their success upon their first discovery of the solution (i.e. their first compound tool construction and use), successful subjects received *replication trials* (max. 3 trials per day), until they had succeeded in 3 or had failed in 6 consecutive replications.

3. *Transfer test 2 (ground):*

The 4 individuals who succeeded in creating compound tools and passed the replication trials in transfer test 1 (Tumulte, Mango, Tabou and Jungle) proceeded to the ergonomically more demanding transfer test 2 (described above). The trials lasted max. 12 min and each bird received at least 8 trials.

4. *Need discrimination test:*

The successful group (including the subject that failed transfer test 2) participated in a further experiment that consisted of 2 preparatory phases and a test.

a) *Preparatory phase I*

The subjects were provided with 4 short dowels (8cm long) and the modified test box with the additional 'close' food track and opening at ca. 6cm from the box's front (figure S5). In this phase the bait could be reached with a short dowel, to make the birds experience the task with food closer than before. Each bird had to pass 6 such trials. If birds succeeded before 12 min has lapsed, the test box was re-baited up to 2 more times.

b) *Preparatory phase II*

In this phase we provided the birds with 4 short and 4 long dowels (13cm long) on the ground. Four short dowels were placed in front of the test box, 2 on each side. Two long dowels were put further away from the baited box than the short dowels so as to increase the effort of picking them up. Notice that in this test long tools worked for both the *close* and *distant* tracks.

The position of the food varied in a counterbalanced and pseudo-randomized order between the *close* and the *distant track*, across trials, 12 times in each position. Hence, birds experienced that the position of the bait could vary across trials, and what length of tool was functional in each case, but did not need to construct compounds in either case. Such discriminations should normally be acquired through general experience.

c) *Need discrimination test*

In this phase, the objective was to test whether the birds combined tools as a response to food being out of reach, i.e. the estimated required tool length. In this case only short (ca. 8cm),

combinable tool elements were presented. These elements were sufficiently long -without combining- to reach food in the close track, but food placed in the distant track could only be reached by making compound tools.

Eight sessions of 3 trials each were conducted in which the position of the bait varied pseudo-randomly between the *close* and the *distant* track, testing the birds with each position 12 times.

5. *Construction test 2 (three-compound tool)*

Birds that had succeeded in all previous transfer tasks (i.e. Mango, Tabou and Jungle) were provided with tool elements so short (4,5-5cm) that a tool constructed of 2 such elements was not sufficient to reach the food in the track at 12 cm distance from the box's front (figure S6). Functional tools could be made by combining 3 tool elements, either using a syringe tube with a dowel at either end, or a dowel with a syringe tube at either end. Twelve trials of max.12min each were conducted.

4. Scoring and Analysis.

Video analyses were carried out to score individual performance, frequency of combinatory attempts (trying to insert a tool element into the opening of another, or inserting a tool element into another, but without succeeding to create a combined tool sufficiently stable to be lifted) and interaction time with tool elements (time in sec an individual spent manipulating tool elements until first success). A success was scored if a subject created a sufficiently stable combined tool, inserted it in the test box, and retrieved the food. The data were scored by two raters independently and they interpreted the same instances as compound tool construction. The inter-observer reliability was assessed for the interaction time until first success and the Pearson's correlation coefficient was more than 99% (Pearson's correlation coefficient: $r = .993$, $p < .001$). The time was taken to the nearest second.

For the need discrimination test, where food was located either within or beyond the reach of uncombined single tool elements, we examined whether the birds first inserted a combined or an uncombined tool into the test box in each of the 12 trials of both conditions (*close* and *distant* track), and also looked at whether individuals successfully retrieved the food with a combined or uncombined tool. Because we had only 4 subjects for this test, we evaluated the statistical reliability of the results with Fisher Exact tests at individual level.

B. ADDITIONAL RESULTS.

Detailed information on individual performance in terms of trial duration until first success and number of combinatory attempts with tool elements before first success in each test condition can be found in tables S1-S2. In the following we provide additional information in the form of qualitative descriptions of the behavior of the individual subjects. The performance in replication trials is reported.

1. Construction test and replication trials

Tabou, Jungle and Tumulte succeeded in the 3 trials following their first successful trial. Tumulte could not retrieve the food in her very first replication trial although she had

successfully combined a tool, because of an experimental error.[†] We considered this trial as successfully passed, but gave her 3 additional trials, in which she also retrieved the food consistently. Mango, an individual whose motivation appeared to be highly variable, did not combine elements in the first replication trial, then successfully combined them in the second and the third trials but turned its attention elsewhere and did not retrieve the bait. He successfully combined elements and retrieved the reward in the following 3 replications.

2. Transfer test 1 (straw) and replication trials

Individual performance is detailed in tables S1-S3. In descriptive form: Tumulte combined a dowel and a straw in her first straw transfer trial and Mango in his second trial (both without any previous combinatory actions or attempts). Tabou succeeded in her fourth trial (with just a single prior combinatory attempt) and Jungle (who seemingly exhibited motivation issues, probably because he had been transferred into a new aviary) created but did not use a combined tool in his first trial and then succeeded in his sixth trial (with one prior combinatory attempt). Tabou and Jungle succeeded in 3 subsequent replications trials right away. Tumulte failed in the first 2 replications but then succeeded in the following ones, and Mango – again because of apparently engagement with the task - failed during the first 3 replications but then succeeded in 3 successive ones.

3. Transfer test 2 (ground) and follow-up trials

Individual performance is given in table S1-S3. In descriptive form: 3 out of 4 subjects succeeded in this second transfer task (Mango, Jungle and Tabou). In their first trial, Jungle and Mango successfully created a new tool (without prior combinatory attempts), Tabou attempted to combine a dowel with a syringe in her first trial but did not succeed and then only tried again and successfully combined and retrieved the food in her third trial (after 2 further combinatory attempts). Tumulte failed the task, apparently due to motoric difficulties and fluctuating motivation towards the task. She made no combinatory attempts in the first 2 trials, but created a combined tool in the third trial. The tool, however, was not stable and fell apart, so that she failed to get the reward and seemed to lose motivation. In subsequent trials she did not engage with the task at all, thus failing the transfer test.

All 3 successful individuals passed the subsequent 3 replication trials.

4. Need discrimination test

Each bird succeeded to retrieve the reward with a combined tool in all 12 trials of the *distant track* condition. This is not, however, direct evidence of them anticipating the need for making compound tools, because they could have resorted to combining after failing with uncombined elements, which only happens in the distant track condition. Yet, in the *close track* condition, where combining was not necessary, the birds only rarely retrieved food with a compound tool, and this only happened in trials in which the first tool inserted was a compound, so that they immediately succeeded. To test directly whether they combined tools as a consequence of perceiving that this was necessary, we compared between the close and distant track conditions

---

[†] The experimenter had failed to check whether the modelling clay in the tool elements was 'functional' and to exchange it by fresh one. The clay had become dry and hard and did not hold the elements together properly. Thus, this replication trial should be considered as 'passed', but –to be conservative- we only accepted the replication success after further 3 trials.

the number of cases in which a compound tool was the first one to be made and inserted, that is before even trying with a single element. The results are shown in figure 2 and table S3.

5. Transfer test 3 (three-component tool) and replication trials

One out of the 3 subjects that had passed transfer test 2 and then proceeded to the third transfer test, Mango, also succeeded to produce tools out of 3 and 4 components. It would appear that for this individual the challenge of making multiple (>2) component tools was ergonomic rather than cognitive. Mango created reasonably stable proto-tools of 2 short elements readily and used them in the test box, but had difficulties adding on the third element with enough force to achieve a tool of sufficient stability; the proto-2-component tool tended to break apart during the process. He made the first combinatory action with a proto-2-component tool and another tool element in trial 1, thus demonstrating sensitivity to the need for the next recursive step. From trial 4 onwards and after a total of 7 combinatory actions in total, he produced 3-component tools that could be lifted. His first 3-component tool (dowel-syringe-dowel) fell apart after he had inserted it in the box and targeted at the food twice. Nevertheless, within the same trial, he created another and targeted it at the food. It initially broke as well, but he managed to fix it, immediately re-inserted it into the slot and finally retrieved the food with it. In trial 5 he succeeded again to produce a 3-component tool and retrieve the food with it, although it broke apart in the end. In trial 6 Mango produced a 3-component and then a 4-component tool inside the box, but neither was sufficiently stable to complete the reward extraction. He finally succeeded to extract food with a 3-component tool in the following trial 7. In subsequent tests he built and used 3-component tools in trial 9 and 10 in order to retrieve the food (but failed to extract the food in trial 9). In trial 6 and 9 he produced a 4-component tool which he used to move the food reward, but which was not fully extracted in trial 6 and got stuck in trial 9. In trial 11 he built another 4-component tool, which he did insert inside the box.

C. FIGURES.

Fig. S1.

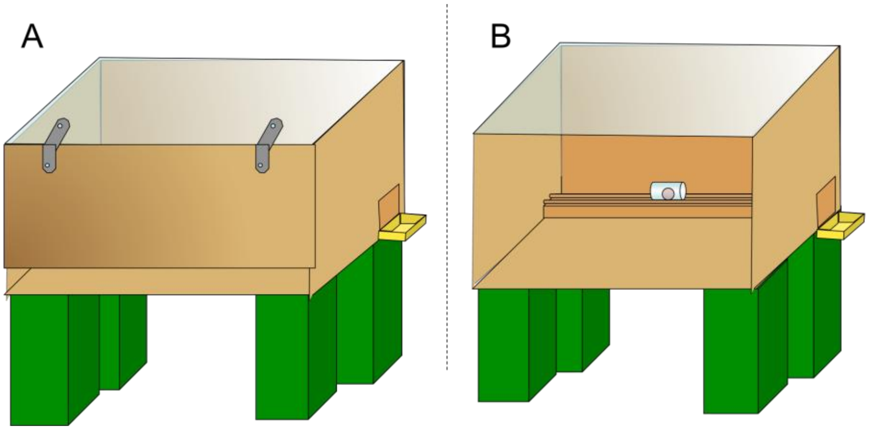

Fig. S1. The test box seen from the front with (A) and without (B) front cover. The test box (14cm x 26cm x 14cm) stood on poles (13cm high) and had a clear Perspex top and a slot for tool insertion on its front side (see A). B shows the box without front cover to reveal its interior: a small plastic container baited with food was placed in the track at the back of the box (B, center of the back side). The subjects could retrieve food by inserting a sufficiently long tool into the

slot in the front of the box (see A) and pushing the plastic container sideways towards an opening at the end of the track. The opening was at either the left or right side of the track according to the individual crow's lateral tool holding bias[‡].

[‡] Weir, A.A.S., Kenward, B., Chappell, J. & Kacelnik, A. Lateralization of tool use in New Caledonian crows (Corvus moneduloides). *Proc. R. Soc. Lond. B* Biol. Sci. **271**(5). S344-S346 (2004).

Fig. S2.

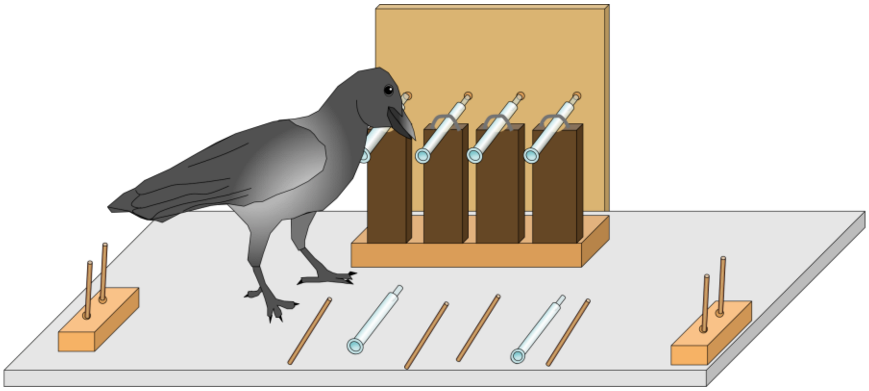

Fig. S2. Tool elements presentation in the construction test 1 (syringe holder). In this construction test potential tool elements were presented on a board with 4 wooden pillars (dark brown, 10.5cm high), each with a furrow on which a syringe (8cm long) could rest, loosely secured by a loop of wire. The tips of the 4 syringes were lightly inserted in holes drilled into a wooden back wall (18.5cm x 39.5cm) behind the pillars, in order to keep them in a horizontal position. Two more syringes were presented loosely on the board, between 4 dowels (8cm long; ca. 0,3cm diameter) spread out in front of the 4 pillars. Four more dowels were presented vertically in 2 wooden holders shown in the left and right edges of the board.

Figure S3.

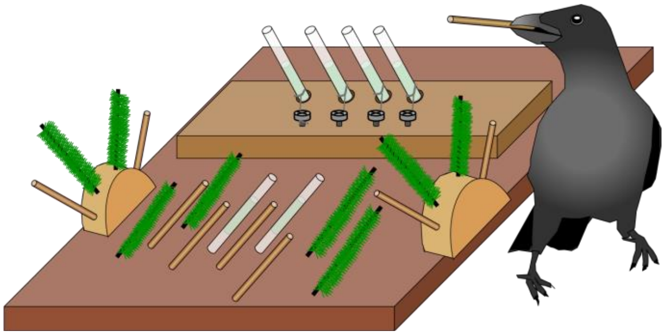

Fig. S3. Tool elements presentation for the transfer test 1 (straw holder). In transfer test 1 tool elements were presented on a board (40cm x 40cm) with a wooden block (31cm x 9cm x 2cm) that had 4 obliquely drilled holes (3cm apart) so that 4 straws were held at a ca. 45° angle towards the center of the board[§]. This allowed the birds to see that the novel straws were hollow inside. The straws were further held in their position by pieces of string as shown. Like in the syringe condition, 2 loose straws were presented on the board between 4 dowels (8cm) and 4 pieces of green pipe cleaner (8cm) spread out in front of the wooden block. Four more dowels and 4 more pipe cleaners were set up in wooden holders at the sides, as shown.

[§] A slightly different version of this tool holder was used initially for the first 3-4 straw trials before it was modified to the version depicted here. In the previous version the straws (which were novel objects to the crows) were presented vertically. We noticed that the birds could not see that the straws were hollow and also would have had difficulties to insert the dowels from above, so we changed the angle to 45° and disregarded those initial trials.

Fig. S4:

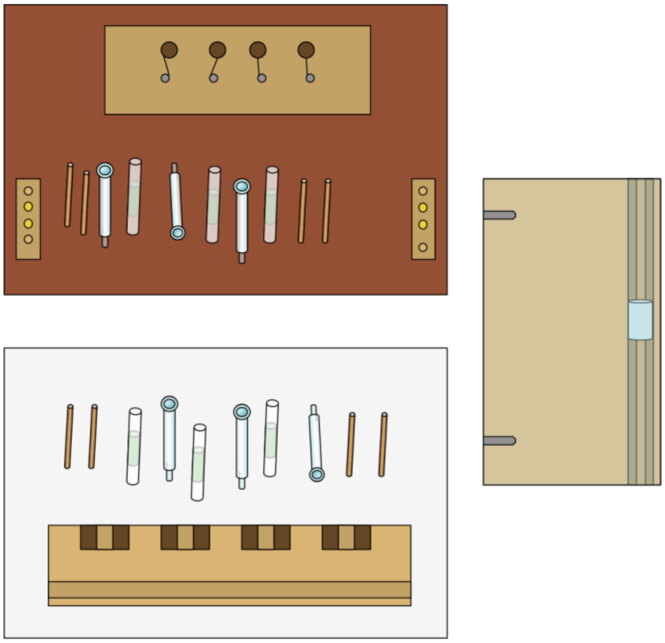

Fig. S4: Top view of the experimental setup of transfer test 2. In transfer test 2, the 2 previously described tool holders were placed opposite to each other (with a ca. 10cm gap between them) and perpendicular to the test box in the center, creating a U-Shape configuration. Both tool holders were empty, but 3 straws and 3 syringes were laid out in alternating order, with 2 dowels on either side (i.e. resulting in a total of 6 straws, 6 syringes and 8 dowels) on each board.

Fig. S5.

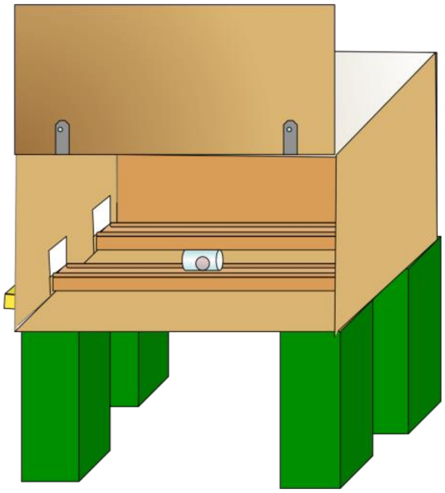

Fig. S5. Test box with the additional 'close track' in the need discrimination test (shown with lifted front cover). An additional *close* food track (shown with the bait) with an extra opening at one end[**] was added to the test box. Food placed in the close track could be reached with individual tool elements, without combining. The original food track is referred to as *distant* track for this control.

[**] The side of the opening varied according to individual side biases due to tool holding lateralisation (see above).

Fig. S6.

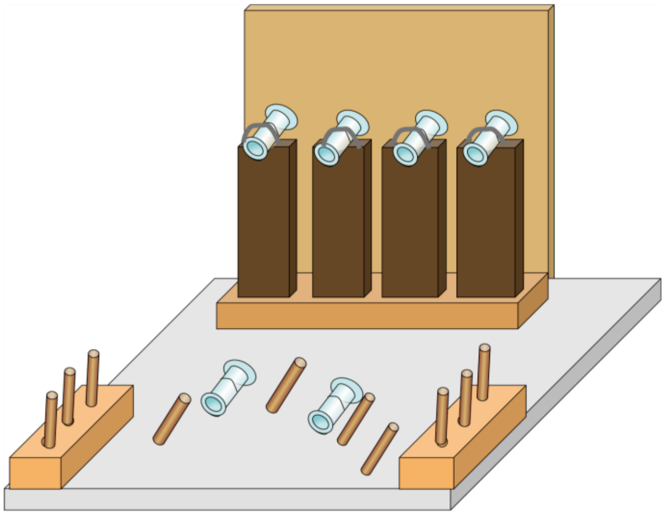

Fig. S6: Setup in construction test 2 (three-component tool). The setup consisted of a tool holder in which 4 short syringe tubes were presented horizontally on wooden pillars. Three short sticks were presented in vertical orientation on each of 2 wooden holders mounted on either side of the board. Four additional short sticks and 2 syringe tubes were presented loosely on the center of the board.

D. TABLES.

Table S1. Latency (trial duration) to first success in the construction test and transfer tests 1 and 2. The trial duration latency (in min:sec) until the first combined tool was created is given for the first successful trial. The time code in brackets indicates the total time across trials within a condition until the first successful trial.

**Subjects**	**Construction test (min:sec)**		**Transfer test 1 (min:sec)**		**Transfer test 2 (min:sec)**	
Jungle	06:32 (30:32)	3	04:19 (62:47 min)	6	01:38 (01:38)	1
Tumulte	00:51 (12:51)	2	01:23 (01:23)	1	/	/
Tabou	01:31 (63:40)	6	00:43 (34:24)	4	01:09 (25:58)	3
Mango	02:50 (53:53)	5	01:16 (13:22)	2	06:05 (06:05)	1

Table S2. Overview of combinatory attempts before the first success in construction test 1 and transfer tests 1 and 2. The second column shows the trial number in which the bird first succeeded to create a combined tool and use it to retrieve the food. For each subject the number of combinatory attempts per trial until the bird succeeded is given in the columns that follow to the right (an X indicates failure in a condition). Trials in which the birds did combinatory attempts are highlighted in color. Trials that were not counted because the birds discovered an unplanned functional hidden tool in their testing chamber (e.g. a hidden long stick) are crossed out. The final number of attempts is summed up in the last column. Here, numbers in brackets show in how many trials the bird had made combinatory attempts. Single asterisks identify combinatory attempts that resulted in an unstable combined tool. Double asterisks indicate cases in which a combined tool was created but not used because the subject ceased to be engaged with the task.

Construction test 1 until first success														
Subject	*1st success*	*1*	*2*	*3*	*4*	*5*	*6*	*7*	*8*	*9*	*10*	*11*	*12*	*No. of attempts (att. trials)*
Tumulte	**2**	0	0											0 (0)
Tabou	**6**	0	0	1*	0	1**	1							3 (3)
Mango	**5**	7**	3		0	0	0							10 (2)
Jungle	**3**	2	5	0										7 (2)
Transfer test 1 (straw) until first success														
Tumulte	**1**		0											0 (0)
Tabou	**4**	0		1	0	0								1 (1)
Mango	**2**		0	0										0 (0)
Jungle	**6**	1**	0	1	0	0	0							2 (2)
Transfer test 2 (ground) until first success														
Tumulte	**(3)**	0	0	4*										4 (1)
Tabou	**3**	1	0	4										5 (2)
Mango	**1**	0												0 (0)
Jungle	**1**	0												0 (0)

Table S3. Performance in the control test.

Number of trials in which the birds created a combined in the critical control condition *close track,* where the reward could be reached with a short dowel or in the baseline condition *distant track* where the reward could only be reached only with a combined tool. Asterices indicate performance above chance (binomial probabilities).

	***close track* - critical**	***distant track* - baseline**
**Jungle**	5/12	12/12*
**Tumulte**	0/12*****	12/12*
**Tabou**	3/12*****	12/12*
**Mango**	6/12	12/12*

E. MOVIES.

Still Image Movie S1.

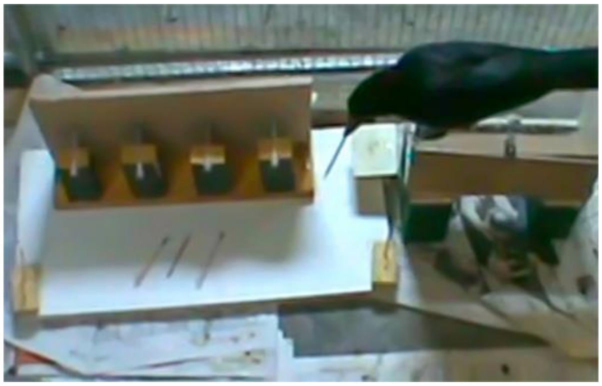

Movie S1. Construction test 1: First success of the subject 'Tumulte' in trial 2 (after 0:51min). Tumulte lands in front of the test box and approaches it, on the way collecting a short dowel from the vertical tool holder (this action is obscured by the crow's body) and - holding the tool element but not inserting it - looks inside the slot of the test box. Next, she lands on top of the box with the dowel still in her beak and looks inside. She then hops from the box onto the platform in front of the horizontal tool holder, inserts the dowel into a syringe on the holder, carefully pulls the combined tool out of the tool holder, inserts it into the box and retrieves the food reward.

Still Image Movie S2.

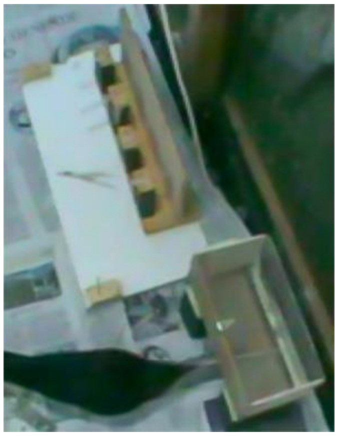

Movie S2. Construction test 1: First success of the subject 'Jungle' in trial 3 (after 6:32min). Jungle picks up a dowel from the platform in front of the horizontal tool holder, inserts it in a syringe on the tool holder, and carefully pulls the combined tool off the holder. Straight afterwards he walks towards the box and inserts the newly created compound tool in the slot and retrieves the food reward.

Still Image Movie S3.

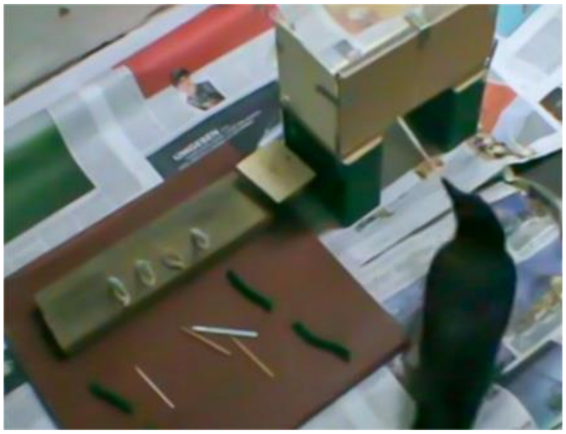

Movie S3. Transfer test 1 (straw). First success of the subject ‘Tumulte’ in trial 1 (after 1:23 min). Tumulte lands briefly to collect a dowel and 64 seconds later she again approaches the tool holder with the dowel in her beak directing it towards the straws in the holder. She inserts the dowel and pulls the combined tool off the holder. Afterwards she approaches the box, initially aiming towards the exit hole for the food reward but then switching to target the front slot and correctly inserting the combined tool.

Still Image Movie S4.

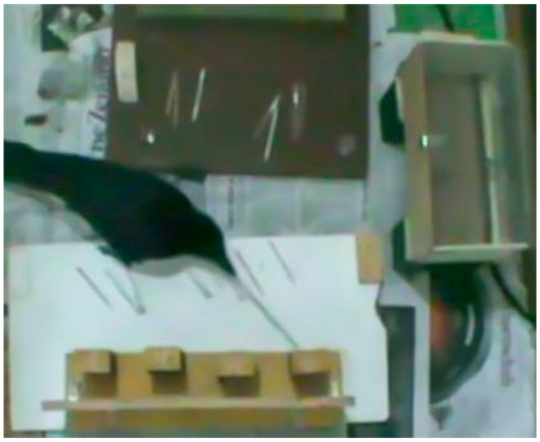

Movie S4. Transfer test 2 (ground): First success of the subject ‘Jungle’ in trial 1 (after 1:38 min). Jungle picks up a short dowel from the platform and inserts it into a syringe on the ground. While inserting, the syringe slides away across the platform, but Jungle pushes it against the bottom of the tool holder and thus succeeds to create a functional compound tool. Afterwards, he immediately inserts the tool into the slot of the test box and retrieves the food reward.

Still Image Movie S5.

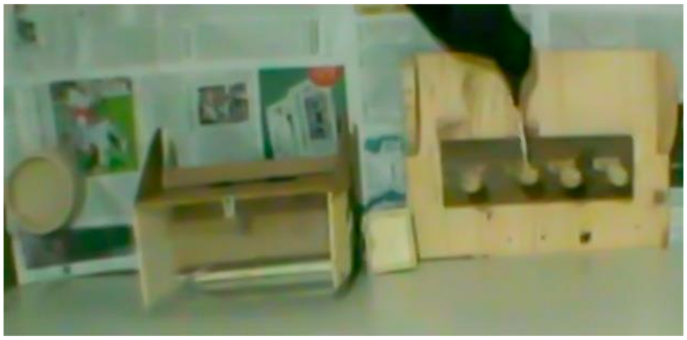

Movie S5. Construction test 2: Fifth success of subject 'Mango' in creating and using a 3-component tool in trial 9 (after 6:50 min). Initially Mango collects a dowel from the tool holder and uses it to explore the ground underneath the food reward platform on the side of the box. Afterwards, he pushes the dowel against a 2-component tool on the ground and combines a 3-component tool by pushing all elements against the side of the wall. He inserts the tool briefly inside the box and then pushes it against the tool holder. Afterwards, he again inserts the tool inside the box and targets the food rewards in the left corner.

Still Image Movie S6.

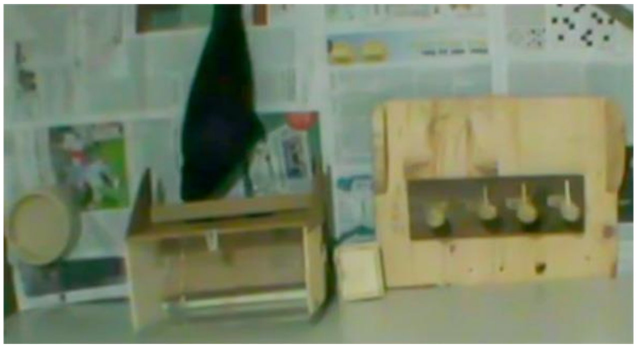

Movie S6. Construction test 2: Creation of a 4-component tool by the subject 'Mango' in trial 9 (after 3:30 min). Mango collects from the ground a previously combined 3-component tool and approaches the slot of the box. He inserts the tool into a syringe tube piece that he had previously inserted in the test box. He manages to push the food reward (and the other loose syringe tube) to the left corner of the food track and then keeps trying to direct the reward out of the box. However, the reward becomes stuck and finally the 4-component tool falls apart.
